# Intracranial mesenchymal tumor with FET::CREB fusion: a rare case report

**DOI:** 10.3389/fonc.2026.1808296

**Published:** 2026-06-18

**Authors:** Weina Ma, Tianping Li, Yahui Yi

**Affiliations:** 1 Department of Ward 505, The First People’s Hospital of Jiashan County, Jiaxing, Zhejiang, China; 2 Department of Radiology, the Second Affiliated Hospital of Jiaxing University, Jiaxing, Zhejiang, China

**Keywords:** case report, CNS tumors, FET::CREB fusion, intracranial mesenchymal tumor, molecular pathology

## Abstract

**Purpose:**

This case report aims to enhance awareness and understanding of intracranial mesenchymal tumors with FET::CREB fusion by illustrating the associated diagnostic challenges, thereby contributing to the limited literature on this rare entity.

**Methods:**

This report presents a case of a 58-year-old female who presented with a persistent headache. Neuroimaging revealed a well-defined cystic mass in the right temporoparietal lobe. The patient underwent gross total resection (GTR).

**Results:**

Histopathological analysis was consistent with an intracranial mesenchymal tumor, showing immunopositivity for Desmin, MUC4, CD99, and ALK. Next-generation sequencing identified an EWSR1::ATF1 gene fusion, confirming the diagnosis of an intracranial mesenchymal tumor, FET::CREB fusion-positive. Approximately two years postoperatively, follow-up imaging revealed local tumor recurrence, which was managed with a second GTR.

**Conclusion:**

This case highlights the diagnostic challenges, potential for recurrence, and importance of molecular profiling in the accurate diagnosis of this rare tumor entity. GTR remains the primary treatment, though the long-term biological behavior and optimal management strategies require further investigation.

## Introduction

The FET::CREB fusion-positive intracranial mesenchymal tumor is an extremely rare intracranial tumor. Previously referred to as intracranial angiomatoid fibrous histiocytoma (AFH) or intracranial myxoid mesenchymal tumor (IMMT), it primarily involves fusions between FET (including EWSR1 and FUS) and CREB family genes (e.g., ATF1, CREB1, and CREM) (1). It was recognized as a provisional entity in the 2021 World Health Organization (WHO) Classification of Tumors of the Central Nervous System (CNS) (2). This paper reports a rare and unique case of FET::CREB fusion-positive intracranial mesenchymal tumor occurring in the temporoparietal lobe of an adult patient, which is notable for a prior misdiagnosis and postoperative recurrence.

## Case presentation

**Figure 1:**
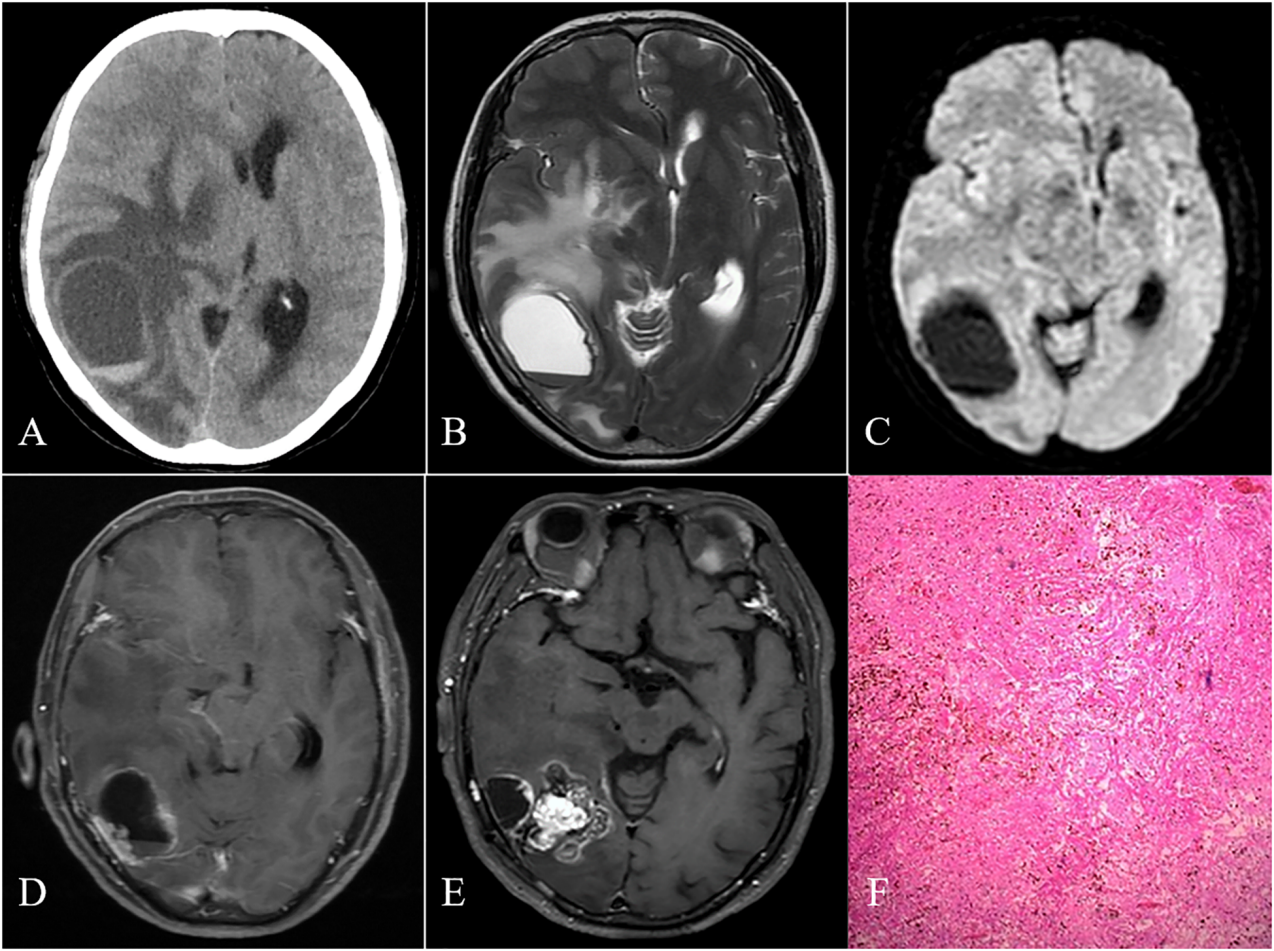
**(A-D)** showed the initial imaging findings of a cystic-solid mass in the right temporoparietal lobe. **(E)**, tumor recurrence at 24 months after surgery. **(F)**, Postoperative pathological examination with HE staining.

A 58-year-old female patient was admitted to the hospital due to a persistent headache for 3 weeks without an obvious cause. A neurological examination performed at admission revealed no abnormalities. Computed Tomography (CT) showed a well-defined 5.1cm × 3.2cm cystic mass in the right temporoparietal lobe, containing hyperdense fluid (**Figure 1A**). Brain MRI demonstrated a mass with heterogeneous cystic fluid signal, unrestricted diffusion, and enhancing cyst walls. Surrounding vasogenic edema involved the posterior frontal, temporal, parietal, and occipital lobes, with a 16-mm leftward midline shift (**Figures 1B–D**). Based on imaging features, both cystic glioblastoma and cystic meningioma were considered in the differential diagnosis. The initial preoperative diagnosis was cystic glioblastoma, which proved to be incorrect on final pathology.

## Management and histopathological findings

The patient underwent right temporoparietal craniotomy with gross total tumor resection. Intraoperative findings revealed a well-demarcated, predominantly cystic mass with solid components and moderate vascularity. Histopathological findings were consistent with an intracranial mesenchymal tumor (**Figure 1F**). The tumor cells demonstrated diffuse strong positivity for Desmin, MUC4, CD99, and ALK (D5F3); positivity for Synaptophysin and EMA; and focal positivity for CD68. Next-generation sequencing (NGS) subsequently revealed an EWSR1::ATF1 gene fusion, along with NKX2–1 p.A69E and SOX17 p.W78* mutations (**Table 1**). The histologic features and molecular alterations support the diagnosis of an intracranial mesenchymal tumor with FET::CREB fusion according to the 2021 WHO classification of CNS tumors. Immediate postoperative MRI showed no residual tumor. Routine surveillance with MRI every six months was recommended.

**Table 1:** Gene alterations detected by next generation sequencing.

Gene	Transcript ID	DNA variant	Aminoacid change	Exonic position	Variant type	MAF/CNA
EWSR1-ATF1	/	/	/	exon7 exon4	Fusion	20.07%
NKX2-1	NM_00107966 8.3	c.206C>A	p.A69E	exon2	Missense	4.39%
SOX17	NM_022454.4	c.234G>A	p.W78*	exon1	Nonsense	3.71%

*MAF, Mutant Allele Frequency; CAN, Copy Number Alteration*.

## Outcome and follow-up

Approximately 24 months after initial surgery, the patient presented again with persistent headache. Brain MRI performed at that time revealed a 5-cm enhancing mass in the right temporoparietal lobe at the surgical bed (**Figure 1E**), indicating tumor recurrence. No interval imaging had been obtained between the immediate postoperative scan and this 24-month scan. Upon detection of the recurrent lesion, the patient underwent a second gross total resection (GTR) without a period of observation, given the symptomatic nature of the mass and the known risk of progression. Pathologic findings were consistent with the initial diagnosis.

After the second resection, adjuvant radiotherapy and chemotherapy were discussed with the patient, but they could not be administered because of the financial constraints. The patient was also unable to attend any further radiological follow-up visits. Consequently, no postoperative imaging is available for the period following the second resection.

## Discussion

FET::CREB fusion-positive neoplasms have been identified recurrently in extracranial AFH, hyalinizing clear cell carcinoma of the salivary gland, clear cell sarcoma of the gastrointestinal tract, clear cell sarcoma of soft tissue, and primary pulmonary myxoid sarcoma (3–7). It was first reported by Dunham et al. (8) in 2008. Intracranial FET::CREB fusion-positive lesions are extremely uncommon, and the relevant diagnostic criteria remain controversial—specifically, whether this condition represents a new entity or a variant of intracranial myxoid AFH (9). These tumors frequently show myxoid and/or fibromyxoid stroma, diverse architectures (e.g., cord-like or sheet-like), and variable cytomorphologies (including epithelioid, rhabdoid, and spindle cells). A subset of intracranial mesenchymal tumors share histopathological and molecular features with AFH or its myxoid variant, yet exhibit a markedly higher prevalence of CREM fusions. Sloan et al. (10) investigated 20 patients with intracranial mesenchymal tumors confirmed by NGS to harbor FET::CREB fusions and proposed the diagnostic term “intracranial mesenchymal tumor, FET::CREB fusion-positive type” for this entity. Previously known as intracranial AFH or IMMT, this tumor is characterized by rearrangements involving a FET family gene (EWSR1 or FUS) with a member of the CREB family of transcription factors (CREB1, ATF1, or CREM) (11). It is currently listed as a provisional entity in the WHO classification of CMS tumors.

Intracranial mesenchymal tumors with FET::CREB fusion are nonmeningothelial mesenchymal neoplasms with a broad morphological spectrum (11). These tumors show a wide age distribution, with a median age of 24 years (range, 4–71 years) and a female predominance (12). The majority of reported cases occur in children and young adults, with approximately 60% of patients under 30 years of age (10, 12). They are commonly extra-axial, supratentorial, intraventricular, or attached to dura or meninges (10). Clinical manifestations depend on tumor location, size, and growth rate, typically including headache, seizures, and signs of elevated intracranial pressure. Although the FET::CREB fusion is a diagnostic hallmark, radiological and histological features can still be misleading. Several reported cases were initially diagnosed as meningiomas based on morphology and only confirmed as FET::CREB fusion-positive tumors by NGS (13–15). On MRI, FET::CREB fusion-positive tumors are well-circumscribed, demonstrate homogeneous or heterogeneous signal intensity, and often enhance markedly. Cystic change and peritumoral edema may occur. In summary, the differential diagnosis for a cystic enhancing intracranial mass with dural attachment includes ependymoma, epidermoid cyst, solitary fibrous tumor/hemangiopericytoma, primary CNS lymphoma, and other CNS tumors (11). In the present case, these entities were effectively excluded by integrated histopathological, molecular, and imaging findings. Immunohistochemistry demonstrated diffuse strong positivity for desmin, MUC4, CD99, and ALK (D5F3), a staining pattern not characteristic of any of the aforementioned tumors. Next−generation sequencing identified an EWSR1::ATF1 fusion, the molecular hallmark of FET::CREB fusion−positive mesenchymal tumors, which is absent in the other differentials. Imaging revealed a well−circumscribed cystic mass with enhancing walls and dural attachment; however, the absence of a dural tail sign and heterogeneous cystic fluid signal made meningioma unlikely, while the lack of ring−like irregular enhancement and significant peritumoral necrosis argued against high−grade astrocytoma. Collectively, these findings support the diagnosis of an intracranial mesenchymal tumor with FET::CREB fusion.

Intracranial mesenchymal neoplasms harboring FET::CREB fusions constitute a rare tumor entity. The clinical prognosis of this tumor is highly variable. Some cases exhibit rapid tumor growth, a propensity for local recurrence or progression, and even distant metastasis. Emerging evidence suggests that FET::CREB fusion-positive neoplasms can be classified into two epigenetic subtypes with significant prognostic implications (12). Additional studies have demonstrated that intracranial mesenchymal tumors harboring the EWSR1::ATF1 fusion are associated with a worse prognosis (10). However, whether distinct gene fusion types or epigenetic subtypes correlate with prognostic differences remains unclear, warranting further investigation through larger case series. GTR is currently considered the treatment of choice; however, recurrence or metastasis may still occur after GTR (16). For recurrent or unresectable tumors, postoperative craniospinal irradiation and chemotherapy are recommended, although the efficacy of chemotherapy remains to be further investigated (17, 18).

Despite GTR, MRI performed after the 24th month following surgery revealed tumor recurrence in this patient, which affected our follow-up strategy. For the current patient, although financial constraints prevented radiological follow-up, we would recommend intensified surveillance if feasible. More generally, for future patients diagnosed with intracranial mesenchymal tumors harboring FET::CREB fusions, we propose: (1) baseline postoperative MRI within 48 hours; (2) close follow-up with MRI every 3-4 months during the first two years post-resection, given the potential for late recurrence; (3) a low threshold for repeat imaging if new neurological symptoms develop; and (4) multidisciplinary discussion for any suspected recurrence, considering early repeat resection rather than prolonged observation.

Beyond structural MRI, molecular imaging modalities—particularly amino-acid PET—merit consideration in the pre-/postoperative evaluation of intracranial mesenchymal tumors with FET::CREB fusion. Although the literature on the PET characteristics of these rare tumors remains extremely limited, one recently published case report has evaluated an intracranial FET::CREB fusion-positive tumor using both ¹^8^F-FDG and ¹^8^F-FET PET/CT (19). Unlike ¹^8^F-FDG, whose high physiological background uptake in normal grey matter often compromises lesion conspicuity, ¹^8^F-FET PET offers high tumor-to-background contrast and is clinically useful for grading intracranial tumors, treatment planning, and distinguishing true progression from treatment-related changes (20, 21). Nevertheless, the extreme rarity of FET::CREB fusion-positive tumors means that systematic data on their PET features are lacking; future case series or multicenter studies are needed to clarify the value of amino-acid PET in this rare entity.

## Conclusion

In conclusion, intracranial mesenchymal tumors with FET::CREB fusion are exceptionally rare, which can be misdiagnosed based on radiological features, and have incompletely characterized biological behavior and long-term prognosis that may vary by genetic fusion subtype (10, 12). Clinical outcomes demonstrate wide heterogeneity. GTR remains the primary treatment objective. In this case, recurrence occurred despite GTR, highlighting the need for close long-term follow-up; however, adherence to surveillance may be limited by patient circumstances. For recurrent or unresectable tumors, adjuvant radiotherapy and/or chemotherapy have been proposed (17, 18), but treatment decisions must consider individual patient circumstances.

## Data Availability

The original contributions presented in the study are included in the article/supplementary material. Further inquiries can be directed to the corresponding author.

## References

[1] Kao YC Sung YS Zhang L Chen CL Vaiyapuri S Rosenblum MK . EWSR1 fusions with CREB family transcription factors define a novel myxoid mesenchymal tumor with predilection for intracranial location. Am J Surg Pathol. (2017) 41:482–90. doi: 10.1097/PAS.0000000000000788 28009602 PMC5350023

[2] Louis DN Perry A Wesseling P Brat DJ Cree IA Figarella-Branger D . The 2021 WHO classification of tumors of the central nervous system: a summary. Neuro Oncol. (2021) 23:1231–51. doi: 10.1093/neuonc/noab106 34185076 PMC8328013

[3] Argani P Harvey I Nielsen GP Takano A Suurmeijer AJH Voltaggio L . EWSR1/FUS–CREB fusions define a distinctive Malignant epithelioid neoplasm with predilection for mesothelial-lined cavities. Mod Pathol. (2020) 33:2233–43. doi: 10.1038/s41379-020-0646-5 32770123 PMC7584759

[4] Antonescu CR Dal Cin P Nafa K Teot LA Surti U Fletcher CD . EWSR1‐CREB1 is the predominant gene fusion in angiomatoid fibrous histiocytoma. Genes Chromosomes Cancer. (2007) 46:1051–60. doi: 10.1002/gcc.20491 17724745

[5] Skálová A Weinreb I Hyrcza M Simpson RHW Laco J Agaimy A . Clear cell myoepithelial carcinoma of salivary glands showing EWSR1 rearrangement: molecular analysis of 94 salivary gland carcinomas with prominent clear cell component. Am J Surg Pathol. (2015) 39:338–48. doi: 10.1097/PAS.0000000000000364 25581728

[6] Desmeules P Joubert P Zhang L Al-Ahmadie HA Fletcher CD Vakiani E . A subset of Malignant mesotheliomas in young adults are associated with recurrent EWSR1/FUS-ATF1 fusions. Am J Surg Pathol. (2017) 41:980–8. doi: 10.1097/PAS.0000000000000864 28505004 PMC5468482

[7] Antonescu CR Katabi N Zhang L Sung YS Seethala RR Jordan RC . EWSR1‐ATF1 fusion is a novel and consistent finding in hyalinizing clear‐cell carcinoma of salivary gland. Genes Chromosomes Cancer. (2011) 50:559–70. doi: 10.1002/gcc.20881 21484932

[8] Dunham C Hussong J Seiff M Pfeifer J Perry A . Primary intracerebral angiomatoid fibrous histiocytoma: report of a case with a t(12;22)(q13;q12) causing type 1 fusion of the EWS and ATF-1 genes. Am J Surg Pathol. (2008) 32:478–84. doi: 10.1097/PAS.0b013e3181453451 18300800

[9] Bale TA Oviedo A Kozakewich H Giannini C Davineni PK Ligon K . Intracranial myxoid mesenchymal tumors with EWSR1–CREB family gene fusions: myxoid variant of angiomatoid fibrous histiocytoma or novel entity? Brain Pathol. (2017) 28:183–91. doi: 10.1111/bpa.12504 28281318 PMC8028671

[10] Sloan EA Chiang J Villanueva-Meyer JE Alexandrescu S Eschbacher JM Wang W . Intracranial mesenchymal tumor with FET-CREB fusion-a unifying diagnosis for the spectrum of intracranial myxoid mesenchymal tumors and angiomatoid fibrous histiocytoma-like neoplasms. Brain Pathol. (2021) 31:e12918. doi: 10.1111/bpa.12918 33141488 PMC8089120

[11] Tauziède-Espariat A Sievers P Larousserie F Benzakoun J Guillemot D Pierron G . An integrative histopathological and epigenetic characterization of primary intracranial mesenchymal tumors, FET:CREB- fused broadening the spectrum of tumor entities in comparison with their soft tissue counterparts. Brain Pathol. (2021) 32:e13010. doi: 10.1111/bpa.13010 34314078 PMC8713527

[12] Wu Z Rajan S Chung H-J Singh O Dazelle K Abdullaev Z . Intracranial mesenchymal tumor, FET-CREB fusion-positive: an integrative analysis of 81 cases. Neuro Oncol. (2026) 28:939–51. doi: 10.1093/neuonc/noag001 41569358 PMC13128485

[13] Kim NR Kim SI Park JW Park CK Chung CK Choi SH . Brain parenchymal angiomatoid fibrous histiocytoma and spinal myxoid mesenchymal tumor with FET: CREB fusion, a spectrum of the same tumor type. Neuropathology. (2022) 42:257–68. doi: 10.1111/neup.12814 35730186

[14] Domingo RA Vivas-Buitrago T Jentoft M Quinones-Hinojosa A . Intracranial myxoid mesenchymal tumor/myxoid subtype angiomatous fibrous histiocytoma: diagnostic and prognostic challenges. Neurosurgery. (2020) 88:E114–22. doi: 10.1093/neuros/nyaa357 32970137 PMC8133328

[15] Ochalski PG Edinger JT Horowitz MB Stetler WR Murdoch GH Kassam AB . Intracranial angiomatoid fibrous histiocytoma presenting as recurrent multifocal intraparenchymal hemorrhage. J Neurosurg. (2010) 112:978–82. doi: 10.3171/2009.8.Jns081518 19731989

[16] Shaikh ST Hajra D Singh S Nagaraju S El-Maghraby H . Intracranial myxoid mesenchymal tumour with EWSR1-ATF1 fusion sans myxoid stroma – report of a newer entity with brief review of literature. Neurol India. (2022) 70:1639–42. doi: 10.4103/0028-3886.355080 36076673

[17] Lucas B McGovern S Whitehead W Aldave G Mohila C Blessing M . OTHR-08. Clinical management of two pediatric cases of intracranial mesenchymal tumors with FET::CREB fusions. Neuro Oncol. (2024) 26. doi: 10.1093/neuonc/noae064.679

[18] De Los Santos Y Shin D Malnik S Rivera-Zengotita M Tran D Ghiaseddin A . Intracranial myxoid mesenchymal neoplasms with EWSR1 gene rearrangement: report of 2 midline cases with one demonstrating durable response to MET inhibitor monotherapy. Neuro-Oncol Adv. (2021) 3:vdab016. doi: 10.1093/noajnl/vdab016 33738448 PMC7954092

[19] Engkebølle C Elisabeth Engelmann B Scheie D Fugleholm K Law I . Intracranial mesenchymal tumor, FET-CREB fusion positive, evaluated with 18F-FET and 18F-FDG PET/CT. Clin Nucl Med. (2024) 49:892–4. doi: 10.1097/rlu.0000000000005346 38914105

[20] Galldiks N Peplinski J-M Kraft M Lohmann P Werner J-M . The role of amino acid PET in the era of checkpoint inhibitors and targeted therapy for brain tumor treatment. Curr Opin Neurol. (2025) 38:681–7. doi: 10.1097/wco.0000000000001425 40802561

[21] Wang J He S Ran B Dong A Zhao H . Extracranial metastases from FET-CREB fusion-positive intracranial mesenchymal tumor on FDG PET/CT. Clin Nucl Med. (2026) 49(4):892–894. doi: 10.1097/RLU.0000000000006459 41950255

